# Implications of fidelity difference between the leading and the lagging strand of DNA for the acceleration of evolution

**DOI:** 10.3389/fonc.2012.00144

**Published:** 2012-10-16

**Authors:** Mitsuru Furusawa

**Affiliations:** Neo-Morgan Laboratory Incorporated, Biotechnology Research CenterKawasaki, Japan

**Keywords:** evolution, acceleration, leading/lagging strand, biased-mutagenesis, replicore, proofreading, polymerase δ

## Abstract

Without exceptions, genomic DNA of living organisms is replicated using the leading and the lagging strand. In a conventional idea of mutagenesis accompanying DNA replication, mutations are thought to be introduced stochastically and evenly into the two daughter DNAs. Here, however, we hypothesized that the fidelity of the lagging strand is lower than that of the leading strand. Our simulations with a simplified model DNA clearly indicated that, even if mutation rates exceeded the so-called threshold values, an original genotype was guaranteed in the pedigree and, at the same time, the enlargement of diversity was attained with repeated generations. According to our lagging-strand-biased-mutagenesis model, mutator microorganisms were established in which mutations biased to the lagging strand were introduced by deleting the proofreading activity of DNA polymerase. These mutators (“disparity mutators”) grew normally and had a quick and extraordinarily high adaptability against very severe circumstances. From the viewpoint of the fidelity difference between the leading and the lagging strand, the basic conditions for the acceleration of evolution are examined. The plausible molecular mechanism for the faster molecular clocks observed in birds and mammals is discussed, with special reference to the accelerated evolution in the past. Possible applications in different fields are also discussed.

## INTRODUCTION

No evolutionary theory has been proposed so far in which the molecular aspect of DNA replication is considered. The main cause of evolution is thought to be mutations accompanying DNA replication. It has been believed that mutations accompanying DNA replication occur stochastically and evenly. Consequently, it can be said that modern evolutionary theories have been built upon this basic thought.

Genomic DNA in all living organisms is replicated by means of the leading/lagging strand system. Therefore, it seems likely that organisms have to pay a higher cost. This is because considerably more enzymes are required for the lagging-strand synthesis than for the synthesis of the leading strand. Theoretically, DNA can be replicated exclusively using the leading strand. Why has nature chosen such a laborious and high cost system? There might be clear evolutional advantages.

For simplification, a linear DNA with one replication origin (*ori*) at one end, that corresponds to a single replicore in eukaryotes, is used as a model. According to the conventional thought, mutations are introduced stochastically and evenly into the two daughter DNAs. In this situation, if the mutation rate exceeds the so-called threshold value, the population will be extinct before long. Then, this leads to the speculation that organisms might be keeping mutation rates low, and consequently, evolution would advance slowly.

Because of the complexity of the machinery for the lagging-strand synthesis, it can be hypothesized that the fidelity of the lagging strand might be lower than that of the leading strand. Under this strand-biased-mutagenesis, simulations were carried out. Even if average mutation rates thoroughly exceeded the threshold value, an ancestral genotype was guaranteed in the pedigree and, at the same time, an enlargement of diversity was attained with repeated generations. In other words, the model DNA can produce unchanged progeny maintaining the species, and at the same time, higher mutation rates can provide raw material for evolution (Furusawa and Doi, 1992, 1998. These results support the idea that biased-mutagenesis in the lagging strand may result in the acceleration of evolution without accompanying the extinction of the population (Furusawa and Doi, 1998; Furusawa, 1999).

According to the lagging-strand-biased-mutagenesis model (“disparity model”), “disparity-mutator” microorganisms were provided. A mutator of the intestinal bacterium, *Escherichia coli*, with biased-mutagenesis in the lagging strand was provided, and a disparity mutator of budding yeast, *Saccharomyces cerevisiae*, was also established by deleting the proofreading activity of pol δ. These mutator microorganisms grew normally and showed a quick and extraordinarily high adaptability against very severe circumstances (Tanabe et al., 1999; Shimoda et al., 2006). For instance, an *E. coli* mutator was able to grow colonies even in the presence of *saturated* concentrations of different kinds of antibiotics (Tanabe et al., 1999).

In this context, the principle of the acceleration of evolution is discussed. The usefulness of a DNA-type genetic algorithm (GA) with biased-mutagenesis and of disparity mutators of living organisms is also discussed.

## ACCELERATION OF EVOLUTION BY DISPARITY-MUTAGENESIS IN DIGITAL ORGANISMS

### LEADING/LAGGING-STRANDS REPLICATION SYSTEM PROVIDING EVOLUTIONARY ADVANTAGES

**FIGURE 1:**
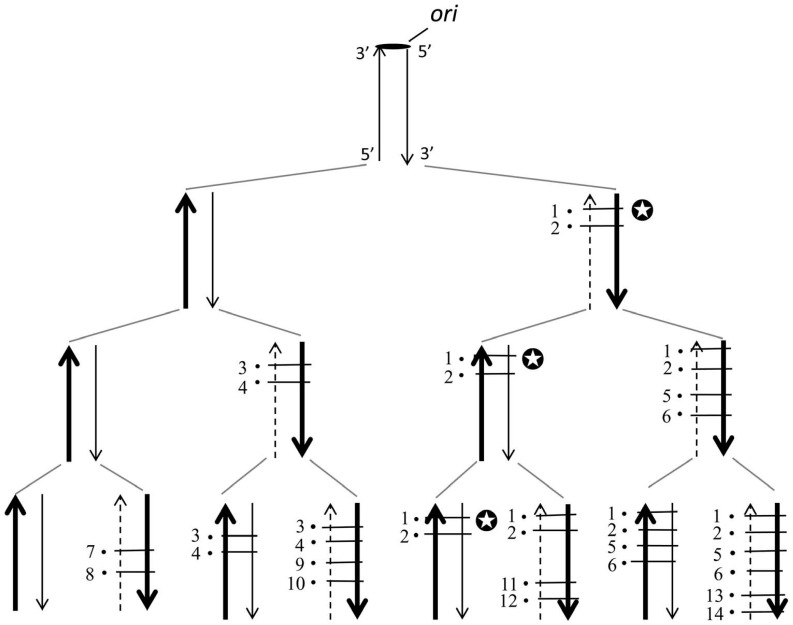
**The distribution of mutations according to the deterministic disparity model of a linear DNA is shown.** A broad arrow indicates a template DNA strand, a thin arrow indicates a newly synthesized leading strand and a dashed thin arrow indicates a newly synthesized lagging strand. The black circle with a short bar crossing strands indicates a point mutation. Each number on the side of a black circle indicates a base substitution at a different site. The *ori* indicates the replication origin. Two mutations per a single replication are introduced exclusively in the lagging strand. Notice that for instance, in the family line of the genomes with the symbol mark (✪), the genotype is guaranteed forever.

**Figure 1** shows a pedigree of a linear chromosomal DNA with a single *ori* at its upper end, i.e., corresponding to a single replicore in higher organisms. It is roughly estimated that several genes, on average, exist in a single replicore. According to our disparity model, biased mutations occur in the lagging strand (Furusawa and Doi, 1992, 1998). The pedigree by the deterministic illustration implies several interesting features; (1) the ancestor has been kept forever; (2) a genotype that once appeared in the past, has been precisely guaranteed in any generation; (3) the threshold of mutation rates is increased (actually, the threshold disappears in this model); (4) even if circumstances changed dramatically, the fittest individual will be selected as a new ancestor and start again to produce a new pedigree as well (**Figure 1**). These outstanding features appear to be beneficial for evolution.

In the conventional model of DNA pedigree, mutagenesis occurs stochastically and evenly in both strands (not shown). Thus, when mutation rates are higher than the threshold value (ca. one mutation/daughter DNA/replication), all individuals would eventually die because of the excess accumulation of deleterious mutations.

**FIGURE 2:**
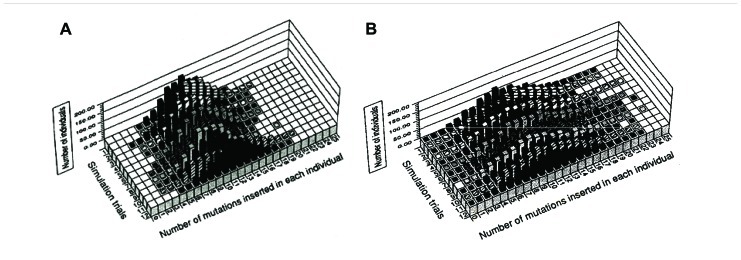
**The distribution of individuals with a given number of mutations in the tenth generation for the parity (A) and disparity** **(B)** stochastic models is shown. The results of 12 trials of simulations are shown. For details see text. Adapted from Furusawa and Doi (1992).

We compared the distribution of mutations in a parity model (average two mutations/replication being introduced into the leading and the lagging strands) with that of a disparity model (average 1.99 mutations in the lagging strand and 0.01 mutations in the leading strand). For the simulation of this stochastic model, a binominal distribution was used. **Figure 2** summarizes the results of 12 trials at the 10th generation. **Figure 2A** shows the distribution of mutants in the parity model. There is no individual with zero-mutations. In contrast, as shown in **Figure 2B**, the disparity model shows a very flat distribution. The ancestral individuals with zero-mutations are always observable except in the ninth trial and highly mutated mutants comparable with the parity model are produced (Furusawa and Doi, 1992). This flat distribution of mutants including ancestral individuals in the disparity model is expected from the pedigree of the deterministic disparity model shown in **Figure 1**.

### DNA-TYPE GENETIC ALGORITHMS WITH BIASED-MUTAGENESIS EFFECTIVELY RESOLVING OPTIMIZATION PROBLEMS

**FIGURE 3:**
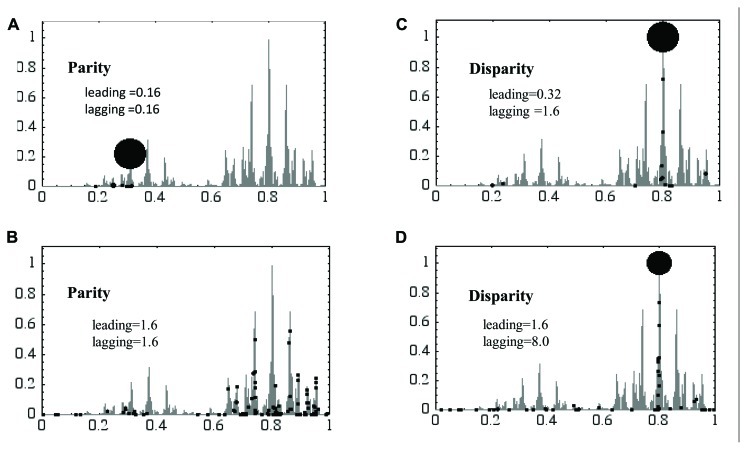
** A “hill-climbing problem” is resolved using the parity and disparity “neo-Darwinian genetic algorithm.”** The genome of the genetic algorithm consists of 16 bit and replicates using the leading and lagging strands. In parity models mutations are introduced evenly in both strands **(A,B)**, while in disparity models biased mutations are introduced in the lagging strand **(C,D)**. The vertical and horizontal axes are the fitness score and the distance of peaks from the starting position, respectively. Black dots symbolize the position of individuals at the end of the experiment. The larger the dots are, the more individuals have accumulated at specific positions. For details see text.

We constructed a GA, named neo-Darwinian algorithm, which consisted of the double-stranded genetic information like DNA (K. Aoki and M. Furusawa, unpublished). This GA replicates using the leading/lagging strands system. We compared the parity model with the disparity model. A simple “hill-climbing problem” with a rugged landscape was provided. The landscape was made using the gray code which has 686 peaks (**Figure 3**). An individual that has reached the foot of a given peak can go up the peak by adding a relatively small number of mutations, while a larger number of mutations are required when an individual moves to another peak at a longer distance.

For simulation, the following conditions were used. The population is always kept at 100, so that 100 individuals having lower fitness scores (FS) have to be omitted from the population after each replication. Each individual has 16 bit, thus the number of genotypes are 2^16^ = 65,536. At the beginning of this game, all 100 individuals are located at the left corner, meaning that all of them have the same genotype with an FS of 0.

**Figure 3** shows the results at the 100th generation. In the parity model, almost all individuals gather around the top of a small hill of low FS at the 0.16 mutation rates (**Figure 3A**). This is because the mutation rate is too low to look for other higher peaks within 100 rounds of replication. In contrast, when the mutation rate goes up to 1.6, all individuals are scattered all around the landscape, meaning that they cannot keep the positions once obtained because of a too high mutation rate (**Figure 3B**). In other words, in the latter case their genetic information has melted away. The disparity model, in which the mutation rate in the leading strand is 0.32 and lagging strand 1.6, showed very nice adaptability. About 90% of the population has reached the top of the highest hill, and the remaining individuals are located elsewhere (**Figure 3C**). The latter ones are wandering around the peaks searching for higher peaks that, however, do not exist. When the mutation rate in the leading strand was increased to 1.6 and the lagging strand to 8.0, about 50% of the population has reached the highest hill. The remaining individuals are broadly located in the landscape searching for higher peaks. The results in **Figure 3** clearly showed how the disparity model has a strong adaptability to severe circumstances, especially when mutation rates are high.

Using a similar neo-Darwinian algorithm, a “knap-sack problem” was resolved. The detailed conditions and the results were shown in our previous report (Wada et al., 1993). The digital individual having two chromosomes is sexually reproduced and its DNA has 100 bit. The number of players is 500. The winner is the individual who collects the best combination of “ores” that have the highest value. When the weight of ores packed in the knap-sack exceeds the weight limitation, his boat sinks, acting as selection pressure.

**FIGURE 4:**
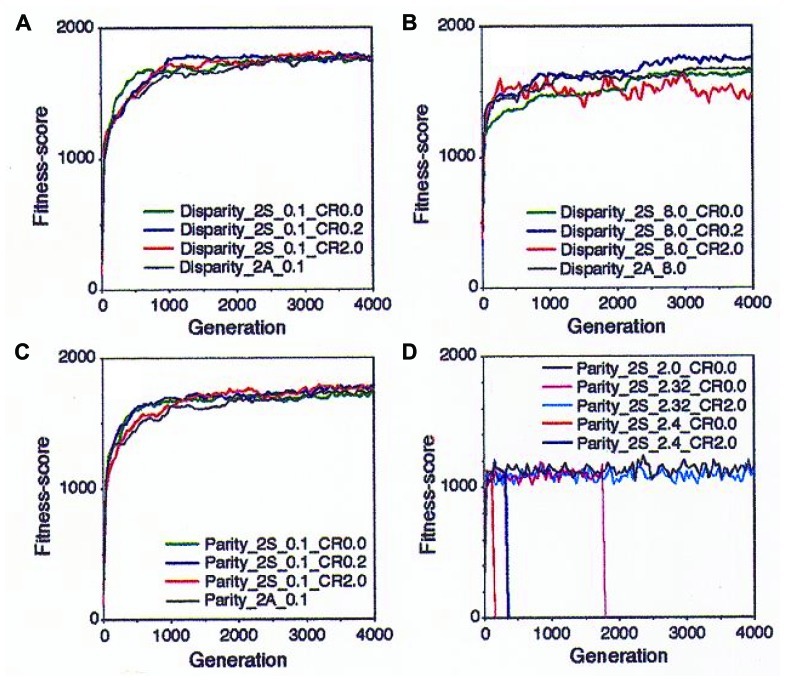
**Results of the simulation with the “neo-Darwinian genetic algorithm” in the diploid and sexual world are shown.** **(A)** Disparity individuals with mutation rate (*n*) = 0.1. Green, no crossover; blue, crossover frequency (CF) = 0.2/chromosome; red, CF = 2.0; and black, asexual and diploid. **(B)** Disparity individuals *n* = 8.0. Green, no crossover: blue, CF = 0.2; red, CF = 2.0; and black, asexual. **(C)** Parity individuals *n* = 0.1. Green, no crossover; blue, CF = 0.2; red, CF = 2.0; and black, asexual. **(D)** Parity individual with various mutation rates. Black, *n* = 2.0 without crossover; magenta, *n* = 2.32 without crossover; cyan, *n* = 2.32 and CF = 2.0; red, *n* = 2.4 without crossover; and blue, *n* = 2.4 and CF = 2.0. Adapted from Wada et al. (1993); Copyright 1993, National Academy of Sciences, USA.

The results are representatively shown in **Figure 4**. At the low mutation rate (0.1), both of the parity and the disparity individuals showed similar results in that they adapted well and kept high FS as far as tested by 4,000 generations (**Figures 4A**,**C**). At higher mutation rates, however, a clear difference was observed between the two models. In the disparity model, even when the mutation rate was as high as 8.0, the individuals showed high adaptability and kept a stable FS. Especially, an appropriate rate of crossover (0.2) increased the FS. In case of the higher crossover rate (2.0), FS was increased with fluctuations. However, the quick increase of the FS curve at early stages of the simulation may mean that the population will quickly occupy the niche, indicating that high crossover rates might act as an advantageous factor for evolution. Individuals with asexual production showed medial FS (**Figure 4B**). When the parity and the disparity individuals competed with each other in the same niche, the latter quickly drove the former away from the niche (data not shown; see Wada et al., 1993).

On the contrary, the parity model showed a very sensitive reaction to small differences of the mutation rates. At the mutation rate of 2.0, the parity individuals showed a constant FS while the FS value was low compared to that of the disparity. When the mutation rate increased to up to 2.32, the population became extinct by the 2,000th generation. The crossover (0.2) dramatically rescued this extinction. When the mutation rate was 2.4, the players were quickly extinct, but crossover delayed the extinction time (**Figure 4D**).

The results of the knap-sack problem are summarized as follows: (1) advantageous conditions for the disparity model: small population, strong selection pressure, high mutation rates, diploid sexual reproduction, and competitive world; (2) advantageous conditions for the parity model: large population, weak selection pressure, low mutation rates, haploid asexual reproduction, and non-competitive world. In conclusion, living organisms, especially when environments change dramatically, might decrease the fidelity of the lagging-strand synthesis in order to be able to better adapt to new environments. After the environment has stabilized, the fidelity might be increased up to the usual level. On the other hand, a parity model might be useful for bacteria or yeasts cultured on an agar-plate with sufficient nutrients (Wada et al., 1993).

### CO-EXISTENCE OF ERROR-PRONE AND ERROR-LESS POLYMERASES ELIMINATING THE EXISTENCE OF ERROR THRESHOLD FROM EIGEN’S “QUASI-SPECIES”

**FIGURE 5:**
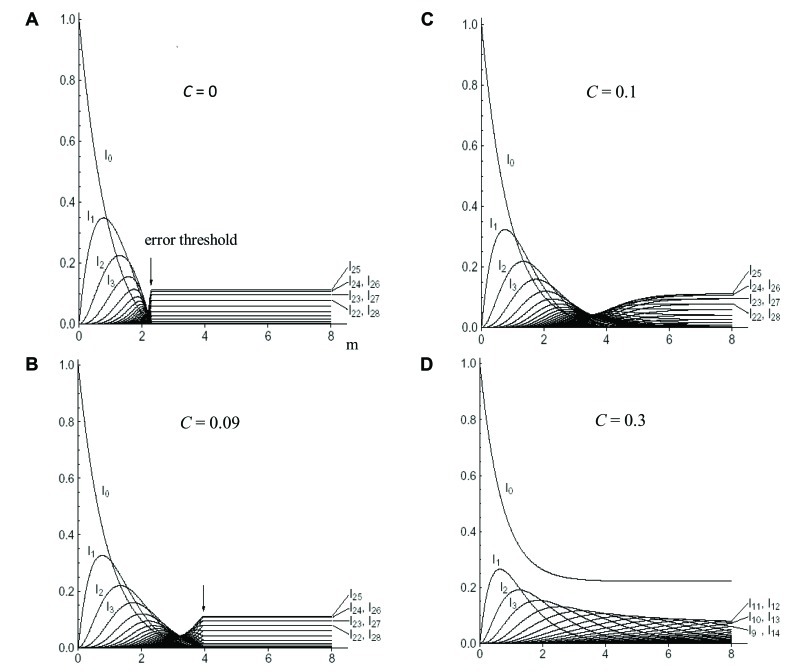
**The mutant distribution in “quasi-species” as a function of the mean error rate (m) per genome.** The genome has a binary base sequence of 50. *c* is the relative concentration of error-free polymerase. The sum of the relative stationary concentration of the wild-type sequence with zero-mutations (I_0_), of all one-error mutants (I_1_), of all two-error mutants (I_2_), etc. are plotted. **(A)** *c* = 0 or the parity model; **(B)** *c* = 0.09; **(C)** *c* = 0.1; and **(D)** *c* = 0.3. Arrow indicates the error threshold. For details see text. Adapted from Aoki and Furusawa (2003); permitted to use this figure from APS Journal, ASP Copyright 2003.

Eigen’s group demonstrated the existence of a critical error threshold using a model RNA (Eigen et al., 1989). Different kinds of RNA polymerase having various fidelities were provided, and each of them was added into a reactor respectively. When the replication reaction reached a stable state, the number of mutations in each RNA molecule was calculated. With increasing error rates of error-prone polymerase, the number of the RNA molecules carrying more mutations naturally increased. Finally, the error rate increased to a critical point just before the genetic information being melted away (the “edge-of-chaos”). If the mutation rate exceeds the critical value, the population immediately falls into a deep chaotic sea, i.e., death (**Figure 5A**).

However, when a mixture of error-less and error-prone polymerases was used, we obtained completely different results. For instance, when a mixture of 9% error-less and 91% error-prone polymerase was used, the position of the error threshold was shifted to the right (**Figure 5B**). When the ratio of error-less polymerase was 10%, the shape of the edge-of-chaos became blunter and the threshold became extinct (**Figure 5C**). When a mixture of 30% error-less and 70% error-prone polymerases was used the extinction of the population was avoided, even if the average mutation rates thoroughly exceeded the threshold value. Of course, the wild-type with zero-mutations existed forever (**Figure 5D**; Aoki and Furusawa, 2003). These effects of the mixture of RNA polymerases with different fidelities can be understood corresponding to the effects of biased-mutagenesis in DNA.

Therefore, it can be predicted from the present result that the efficiency of *in vitro *directed evolution of DNA molecules by error-prone polymerase might be considerably increased by adding error-less and error-prone polymerases simultaneously in the PCR.

## ACCELERATION OF EVOLUTION USING LIVING MICROORGANISMS WITH BIASED-MUTAGENESIS

### *E. Coli* DISPARITY-MUTATOR (*dnaQ49*) ACQUIRING AN EXTRAORDINARILY STRONG TOLERANCE TO ANTIBIOTICS

Based on the results of computer simulations, we tried to accelerate evolution using a mutator strain of *E. coli*, *dnaQ49*. The *dnaQ49 *strain has a temperature-sensitively mutated gene of *dnaQ *encoded 3′–5′ exonuclease (proofreading enzyme). *dnaQ49 *shows high mutation rates at 37°C, but nearly normal mutation rates at 24°C. So, we can control mutation rates at will. We showed that mutations were preferentially introduced into lagging strands and that the average mutation rate was estimated to have significantly exceeded the threshold value. Moreover, the relative mutation rate of the lagging strand was about 100 times higher than that of the leading strand (Iwaki et al., 1996). Irrespective of its high mutation rate, *dnaQ49 *replicates normally. A normal growth rate of *dnaQ49* is the necessary condition for the experimental acceleration of evolution, since DNA replications are absolutely required for evolution.

For the accumulation of mutations, *dnaQ49* was cultured overnight at 37°C without antibiotics, and on the next day it was cultured on an agar-plate containing different concentrations of antibiotics at 24°C. One colony was picked from the plate which contained the highest concentration of antibiotics, followed by a second cycle of mutagenesis. This cycle of mutagenesis/selection procedure was repeated until no colony was formed. The results are summarized in **Table 1**.

**Table 1:** Acquired tolerance of *dnaQ49 *to antibiotics.

	Intact *dnaQ49* MIC (mg/ml)	Acquired tolerance (mg/ml) of *dnaQ49*
Ampicillin	0.002	30^a^ ^b^
Streptomycin	0.001	20.06^a^
Nalidixic acid	0.001	7^a^
Ofloxacin	0.0000156	3^a^

*This table was made using the data from our previous article (Tanabe et al., 1999). MIC, minimum inhibitory concentration*

a Saturated concentration.

b A higher concentration than 30 mg/ml of ampicillin was not available commercially.

Most surprisingly, *dnaQ49 *was able to survive at the *super-saturation* of different kinds of antibiotics tested; ampicillin, streptomycin, and ofloxacin (a derivative of nalidixic acid). The resultant ampicillin-super-resistant *dnaQ49 *that was able to produce colonies at the presence of 30 mg/ml was highly sensitive to other antibiotics, suggesting that the *dnaQ49 *was exclusively adapted to the antibiotics used for the respective selection pressure (**Table 2**). Quinolone is a generic name of a kind of antibiotics including nalidixic acid and ofloxacin, which has anti-gyrase and anti-topoisomerase activity. Quinolone has been clinically used for such a long time that mutant *E. coli *strains have been isolated having acquired tolerance to this drug in patients. Analysis of our ofloxacin-tolerant *dnaQ49 *showed that the sites and the history of point mutations introduced into *gyrA *and *topoIV *genes, which are target genes of quinolone, were coincident with those of the samples from the patients (D83L in *gyrA*, S80R in *topoIV*) Furthermore, no other mutation was found in the limited regions of these two genes as far as sequenced (Tanabe et al., 1999). These results appear to indicate that *E. coli *(*dnaQ49*) evolution is accelerated *in vitro*, and that its results are qualitatively similar to those of naturally occurring evolution.

**Table 2:** Sensitivity of super-ampicillin-tolerant *dnaQ49* to other antibiotics.

	MIC (mg/ml)	
	*dnaQ49*	Super-ampicillintolerant *dnaQ49*
Ampicillin	0.002	2.048^a^^b^
Cefotaxime^b^	0.0000313	0.064
Chloramphenicol	0.001	0.0005
Tetracycline	0.00025	0.00025
Rifampicin	0.008	0.002
Streptomycin	0.001	0.0005
Nalidixic acid	0.001	0.0005
Ofloxacin	0.0000156	0.0000156

*This table was made using the data from our previous article (Tanabe et al., 1999). MIC, minimum inhibitory concentration*

a Growing in the presence of 30,000 µg/ml of ampicillin during prolonged culture time.

b Beta-lactam antibiotic belonging to the group of cephalosporins. Adapted from Tanabe et al. (1999) with minor changes.

Excellent adaptability of another *E. coli* mutator in which the lagging-strand-biased-mutagenesis was introduced by mutated *polI* was reported. As polI ties Okazaki fragments together, the mutated polI introduces mutations exclusively into the lagging strand. When *polI*-mutators having a limited range of mutation rates and having a similar doubling-time were co-cultured, a different mutator strain became the winner of the survival race depending on cases. But, the co-existing wild-type was always driven away from the population (Loh et al., 2010).

**FIGURE 6:**
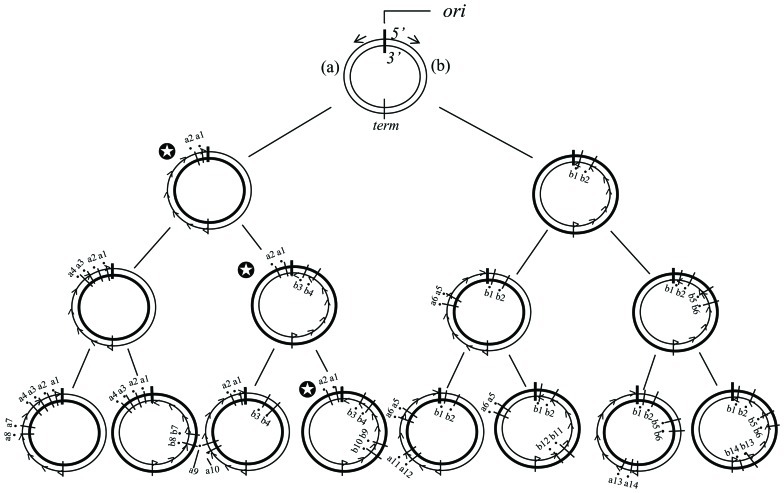
**The distribution of mutations according to the deterministic disparity model of a circular genome is shown.** The *ori* is replication origin. DNA synthesis starts from the *ori* to opposite directions (short arrows). *term*, the position where the progress of DNA synthesis meets. **(a)** and **(b)**, The left and right hemispheres of the genome, respectively. Broad circle indicates the template strand, and thin circle the newly synthesized strand. The thin circle with small arrow heads is the lagging strand. The black circle with a short bar crossing strands is a point mutation. Each number followed by a or b indicates a base substitution at different sites. Two biased mutations are introduced in the lagging strand in every replication. Notice that for instance, in the family line of the genomes with the symbol mark (✪), the genotype of the left hemisphere is guaranteed forever.

**Figure 6** shows the deterministic illustrations of the genotypes in the pedigree of the disparity-mutator of bacteria with a single circular genomic DNA. The presence of a given genotype that has once appeared in the left or right hemisphere of the genome is guaranteed forever. Accordingly, at the left or the right hemisphere of the circular genome, the same phenomenon as shown in the pedigree of the disparity model with a liner DNA can be expected (**Figure 1**). This might explain the reason why the *dnaQ49* mutator showed such a high adaptability against different antibiotics.

### ACCELERATION OF EVOLUTION OF YEASTS BY MEANS OF DISPARITY-MUTAGENESIS

Compared to bacteria, eukaryotic cells such as yeasts have a complex genetic constitution, i.e., a plural number of chromosomes and of *ori*s, a specific life cycle, meiosis, and recombination, etc. Therefore, firstly, we wondered whether a yeast disparity mutator could behave as *E. coli* mutators do.

For the time being, a disparity mutator of the budding yeast *S. cerevisiae* was used, in which the proofreading activity of *polδ *was deleted by two amino acids substitutions. As lagging strands in the yeast are thought to be exclusively synthesized by polδ, this mutator might accumulate mutations in replicores synthesized by mutated polδ. This mutant yeast proliferated as well as wild-type. Fortunately, the results were satisfying for us. We quickly isolated adapted mutants that proliferated well at 40°C and survived at 41°C. Then, in order to stop mutagenesis, the mutated *polδ *gene was replaced by the wild-type one by mating with the wild-type yeast. Genetic analyses indicated that at least two genes were concerned with the temperature-resistant phenotype, and we identified one of them, named *hot1* which contributed to the tolerance against 38.5°C (Shimoda et al., 2006).

Recently, Park and colleagues reported that a disparity-mutator of the filamentous yeast *Ashbya gossypii* showed a ninefold improvement in the production of riboflavin compared to the wild-type strain. This mutant was selected by repeated cloning of cells with higher riboflavin-productivity. To establish the mutator, a plasmid-vector harboring the proofreading activity-deleted *polδ* gene was used for transformation. High riboflavin-producing mutants thus obtained were resistant to oxalic acid and hydrogen peroxide as anti-metabolites. Purine and riboflavin biosynthetic pathways were up-regulated, while pathways related to carbon source assimilation, energy generation, and glycolysis were down-regulated. Genes in the riboflavin biosynthetic pathway were significantly over-expressed (Park et al., 2011; Kato and Park, 2012). Importantly, these phenotypes were stable for a significant period of time.

Using disparity mutators of microorganisms, there have been several reports: (1) improvement of the nature of the yeast cell-wall (Abe et al., 2009a), (2) mutated yeasts having multi-stress tolerance which might be useful, e.g., for bio-ethanol production (Abe et al., 2009b), and (3) improvement of the therapeutic glycoprotein productivity of gene-engineered yeasts, which is toxic for the replication of the host yeast (Abe et al., 2009c), and (4) up-regulation of N_2_O reductase activity in a nitrogen-fixing bacterium (Itakura et al., 2008). We have also established several tolerant *S. cerevisiae *mutants tolerant against pH 2.5, pH 10.3, 2% of isooctane, and 10% of hexane, toluene, chloroform, cyclohexane, and isooctane, respectively (unpublished).

### A STUDY OF MOLECULAR EVOLUTION SUPPORTING OUR DISPARITY MODEL OF EVOLUTION

Katoh et al. (2005) reported that the molecular clock of mammals ran faster than that of other vertebrates. Then, they examined the amino acid substitution rates of polα, polε, and polδ, which mainly contribute to chromosomal DNA replication, obtained from fishes including coelacanth, amphibians, reptiles, birds, and mammals. The result was really exciting for us in that only mammalian *polδ *showed high substitution rates. Moreover, it was recently found out that bird *polδ *also has a higher substitution rate (K. Katoh, personal communication). Thus, it was speculated that the fast run of the bird’s and mammalian molecular clocks might be due to the lower fidelity of *polδ*. More surprisingly, amino acid substitutions occurred intensively in the proofreading (exonuclease)-domain of *polδ*. They also showed that the physicochemical nature of amino acids was changed by those substitutions; strongly suggesting that in the process of bird’s and mammalian evolution, the fidelity of polδ might be occasionally changed. This change in core amino acids for the proofreading activity would cause an occasional up- and down-regulation of the speed of evolution (Katoh et al., 2005). This unexpected coincidence of our experimental results of acceleration of evolution with those findings of Katoh et al. (2005) appears to indicate that *polδ*, especially its proofreading domain, might play a key role for controlling the speed of evolution.

*polδ *may also contribute to lagging-strand synthesis in multi-cellular organisms, though the precise replication mechanism of their genomic DNA is still unclear. In the process of evolution, we can speculate that a similar distribution of mutations shown in **Figure 1** might be displayed in each replicore. Because of the highly complex chromosomal constitutions in eukaryotes, we have not yet simulated the effect of biased-mutagenesis on evolution. There is experimental evidence, however, showing that mouse disparity mutators can normally produce descendents without accompanying severe deleterious phenotypes, though cancer predisposition is increased (Albertson et al., 2009; Uchimura et al., 2009). From these facts, we can imagine that organisms might accelerate evolution by means of decreasing the fidelity of the proofreading activity of *polδ*. Or, at least, we can say that evolution might be experimentally accelerated by deleting the proofreading activity of *polδ*.

Judging from the above-mentioned studies of molecular evolution, it is unlikely that evolution is successfully accelerated by artificially decreasing the fidelity of the polymerase domain of *polδ* because most mutations introduced by the polymerase domain of intact *polδ *may not be introduced at random but at the so-called “hot spots.” Accordingly, artificial impairment of the polymerase domain by gene-manipulation may mean the disturbance of the natural cause of mutations. There is another evidence that supports the advantage of a disparity mutator for the acceleration of evolution; a disparity mutator of *S. cerevisiae* tends to introduce base-changes by transversion with leading to amino acid substitutions while EMS-mutagenesis tends to introduce transitions mainly from G:C to A:T, not leading to amino acid substitutions (Shiwa et al., 2012). However, we should carefully interpret the result of this report. This is because the yeast mutator harboring mutated *polδ* consists of combined mutations of its polymerase- and proofreading-domains.

After all, according to our data discussed here the best target to be manipulated for attaining experimental acceleration of evolution would be the proofreading (3′–5′ exonuclease)-domain of *polδ *gene.

## DISCUSSIONS

### CONCEPT OF DISPARITY MODEL

The most noticeable feature of our disparity-mutagenesis model would be that parental genotypes are guaranteed by error-less leading strands for realizing more reliable inheritance, while lagging strands make a venture for future evolution by accumulating mutations. The acceleration of evolution might be attained by a simple molecular mechanism, i.e., by amino acid substitutions in the proofreading domain of DNA polymerase contributing to the lagging-strand synthesis, that lead to a decrease of the fidelity of lagging-strand synthesis. This mechanism might be shared among all organisms.

Our disparity-mutagenesis model would be directly applicable to viruses and prokaryotes which have a single genomic DNA. This is because template strands are always kept in one daughter cell when replicated. In other words, Cairns’ “immortal strand model” can basically work, in which the “oldest” strand is always kept in the stem cell (Cairns, 1975; **Figure 6**). As eukaryotes, however, have a plural number of chromosomes in each cell and a plural of *ori*s on a single chromosome, situations must be much more complex. The effect of disparity-mutagenesis on eukaryotic evolution remains to be examined.

### EFFECT OF CROSSOVER ON DISPARITY-MUTAGENESIS MODEL

Another point to be considered is that of crossover in sexually reproducing organisms. A limited number of crossovers between each pair of homologous chromosomes occur once during the maturation of germ cells. As shown in **Figure 4**, we can expect appropriate rates of crossover occurring inside a replicore to increase FS. However, usually the crossover might decrease the FS. Anyway, the effect of crossover on FS remains to be examined.

### HIGH MUTATION RATES IN HUMAN AND DISPARITY MODEL

It has been reported that the spontaneous mutation rate of human is inconceivably high, more than 100 mutations/cell/generation. There might be at least 2~3 deleterious mutations which certainly exceed the error threshold (Kondrashov, 1995; Crow, 1997; Drake et al., 1998; Eyre-Walker and Keightley, 1999; Kong et al., 2012). Why have we not died? These excess mutations are believed to be mainly introduced during spermatogenesis (Kong et al., 2012). If we hypothesize that these large numbers of mutations are introduced evenly into the chromosomes of infants, the reason why we are still here would be hard to be explained. Therefore, DNA polymerases that act during spermatogenesis would remain to be analyzed from the viewpoint of disparity-mutagenesis.

### REPLICORE, DISPARITY-MUTAGENESIS, AND EVOLUTION

According to our disparity model of evolution, we can suppose the situation in that a given advantageous replicore once appeared is basically guaranteed in the population for a long time even when mutation rates are high. As if an allele drifts in the population, the replicore as a “stable” genetic unit might be drifting in the population and would be used, by chance, by someone and somewhere. A single replicore usually consists of several genes. Moreover, genes with similar or related functions tend to locate at positions close to each other and/or at a single replicore, e.g., the IgG heavy chain (Brown et al., 1978), β-globin family (Maniatis et al., 1982), *Drosophila *Antennapedia complex (Harding et al., 1985), mouse Hox (Graham et al., 1989), ribosomal RNA (Long and David, 1980), tRNA (Clarkson, 1980), histones genes (Hentschel and Birnstiel, 1981), etc. Thus, the drift of advantageous replicores might offer more drastic effects on evolution than a single gene. Another important condition for guaranteeing the existence of stable replicores in the population would be that germ-lines must have been keeping the positions of *ori*s unchanged for a long time. It seems likely that the existence of the replicore as a stable genetic unit would serve as a main cause of “linkage disequilibrium.”

### APPLICATIONS OF DISPARITY MODEL

DNA-type GA as presented here would be useful for resolving optimization problems with environments changing over time, and the disparity-mutator organisms would be useful not only for the improvement of organisms but also for the improvement of bio-products in medical and industrial fields.

### IMPLICATIONS OF THE ACCELERATION OF EVOLUTION

The concept of artificial acceleration of evolution presented here would serve as a possible tool for opening the door of the black-box existing between gene and organism. Because, we can now see better the process of genomic and phenotypic changes within a living organism (Furusawa, 1999).

### NOVEL BIOLOGICAL FUNCTIONS OF DOUBLE-STRANDED DNA STRUCTURE

Last of all, Cairns’ “immortal strand hypothesis” for cancer prevention (Cairns, 1975), Klar’s “somatic strand-specific imprinting and selective chromatid segregation (SSIS) model” for the determination of differentiation (Klar, 2007), and the present disparity-mutagenesis model would share a common concept. There exists a basic thread common to all of them, i.e., these three lines of research have tried to seek for additional biological implications of the double-stranded structure of the DNA molecule (Furusawa, 2011). Their new paradigm seems to involve implications far beyond conventional molecular biology.

## CONCLUSION

(1) Living organisms are able to represent two contradictory phases; an unchangeable (heredity) and a changeable phase (evolution). According to our disparity theory of evolution, the ultimate cause of the precise heredity would be traced in the leading strand of high fidelity, and that of evolution in the lagging strand of low fidelity.
(2) A double-stranded DNA and its replication machinery can be regarded as a sort of GA. Thus, the fidelity difference between these two strands would act as a factor which increases the resolution ability for optimization problems in environments.
(3) The disparity-mutagenesis model can predict that the speed of evolution appears to be regulated by mutations introduced in the proofreading domain of polδ during the evolutionary process.
(4) Artificially decreasing the proofreading activity of polδ, which may cause increasing the rate of biased-mutagenesis in the lagging strand, would give rise to the experimental acceleration of evolution in eukaryotes.

## Conflict of Interest Statement

The author declares that the research was conducted in the absence of any commercial or financial relationships that could be construed as a potential conflict of interest.

## Acknowledgments

The author thanks Dr F. Rueker for his critical reading of the manuscript and for useful suggestions. The author also thanks Drs A. Uchimura and T. Yagi for their fruitful discussions about high mutation rates in human.
